# Multimodal deep learning integration for predicting renal function outcomes in living donor kidney transplantation: a retrospective cohort study

**DOI:** 10.1097/JS9.0000000000003494

**Published:** 2025-09-17

**Authors:** Jin-Myung Kim, HyoJe Jung, Hye Eun Kwon, Youngmin Ko, Joo Hee Jung, Sung Shin, Young Hoon Kim, Young-Hak Kim, Tae Joon Jun, Hyunwook Kwon

**Affiliations:** a Division of Kidney and Pancreas Transplantation, Department of Surgery, Asan Medical Center, University of Ulsan College of Medicine, Seoul, Republic of Korea; b Department of Information Medicine, Asan Medical Center, Seoul, Republic of Korea; c Division of Cardiology, Department of Internal Medicine, Asan Medical Center, University of Ulsan College of Medicine, Seoul, Republic of Korea; d Department of Medical Informatics and Statistics, Asan Medical Center, University of Ulsan College of Medicine, Seoul, Republic of Korea

**Keywords:** kidney transplant, machine learning, multimodal deep learning, post-transplant outcome prediction, prognosis

## Abstract

**Background:**

Accurately predicting post-transplant renal function is essential for optimizing donor-recipient matching and improving long-term outcomes in kidney transplantation (KT). Traditional models using only structured clinical data often fail to account for complex biological and anatomical factors. This study aimed to develop and validate a multimodal deep learning model that integrates computed tomography (CT) imaging, radiology report text, and structured clinical variables to predict 1-year estimated glomerular filtration rate (eGFR) in living donor kidney transplantation (LDKT) recipients.

**Materials and Methods:**

A retrospective cohort of 1,937 LDKT recipients was selected from 3772 KT cases. Exclusions included deceased donor KT, immunologic high-risk recipients (*n* = 304), missing CT imaging, early graft complications, and anatomical abnormalities. eGFR at 1 year post-transplant was classified into four categories: >90, 75–90, 60–75, and 45–60 mL/min/1.73 m^2^. Radiology reports were embedded using BioBERT, while CT videos were encoded using a CLIP-based visual extractor. These were fused with structured clinical features and input into ensemble classifiers including XGBoost. Model performance was evaluated using cross-validation and SHapley Additive exPlanations (SHAP) analysis.

**Results:**

The full multimodal model achieved a macro F1 score of 0.675, micro F1 score of 0.704, and weighted F1 score of 0.698 – substantially outperforming the clinical-only model (macro F1 = 0.292). CT imaging contributed more than text data (clinical + CT macro F1 = 0.651; clinical + text = 0.486). The model showed highest accuracy in the >90 (F1 = 0.7773) and 60–75 (F1 = 0.7303) categories. SHAP analysis identified donor age, BMI, and donor sex as key predictors. Dimensionality reduction confirmed internal feature validity.

**Conclusion:**

Multimodal deep learning integrating clinical, imaging, and textual data enhances prediction of post-transplant renal function. This framework offers a robust and interpretable approach for individualized risk stratification in LDKT, supporting precision medicine in transplantation.

**HIGHLIGHTS**

- Developed a multimodal deep learning model to predict 1-year renal function after living donor kidney transplantation (LDKT).
- Integrated structured clinical data, radiology report text (BioBERT), and CT video features (CLIP) for eGFR prediction.
- Multimodal model outperformed unimodal models, achieving a macro F1 score of 0.675 vs. 0.292 for clinical-only input.
- CT imaging provided greater predictive value than radiology text when combined with clinical data.
- SHAP analysis revealed donor age, BMI, and donor sex as the most influential predictors of graft function.
- Embedding visualization with t-SNE and UMAP demonstrated clustering patterns across eGFR classes.
- Model offers an interpretable and robust framework for individualized graft function prediction and transplant planning.

## Introduction

Kidney transplantation (KT) is the preferred renal replacement therapy for patients with end-stage renal disease (ESRD), offering superior long-term survival and improved quality of life compared to dialysis^[1–5]^. Despite advances in surgical techniques and immunosuppression, predicting long-term graft function remains a significant clinical challenge. In particular, estimation of post-transplant glomerular filtration rate (GFR), a key surrogate marker for graft health and recipient prognosis, is critical for guiding clinical decision-making, optimizing donor-recipient matching, and tailoring postoperative management strategies^[6,7]^.

Traditional models for predicting renal graft function have primarily relied on structured clinical parameters such as donor age, body mass index (BMI), human leukocyte antigen (HLA) mismatch, and recipient comorbidities^[8–10]^. While informative, these approaches often fail to capture the complex, high-dimensional interactions among biological, anatomical, and perioperative variables^[11,12]^. Consequently, there is a growing need for predictive frameworks that leverage the rich, multimodal data available in contemporary clinical practice, including radiological imaging and unstructured textual information from clinical reports.

Recent developments in artificial intelligence (AI), and deep learning in particular, have shown considerable promise in various domains of medical informatics^[13–17]^. Convolutional neural networks (CNNs) and transformer-based architectures have enabled automated feature extraction from imaging and text data^[18,19]^, while ensemble learning models such as eXtreme Gradient Boosting (XGBoost)^[20,21]^ and Random Forests have demonstrated moderate performance on structured clinical data^[22,23]^. Within the domain of KT, prior machine learning studies have predominantly focused on short-term outcomes such as delayed graft function (DGF) or acute rejection^[24–28]^ with limited work addressing personalized, long-term functional outcomes such as post-transplant GFR. Recent efforts have demonstrated the potential of machine learning models for predicting transplant outcomes using structured preoperative data^[29,30]^. For instance, Ali et al^[31]^. developed the Live-Donor Kidney Transplant Outcome Prediction (L-TOP) model using deep Cox mixtures to accurately predict graft survival in living-donor transplant recipients, outperforming conventional tools across subgroups and timepoints. However, prior models have largely relied on tabular inputs, without incorporating unstructured text or image modalities. Our study addresses this methodological gap by integrating structured clinical data, radiology report embeddings, and CT imaging features into a unified predictive model for early post-transplant eGFR.

Unimodal models that rely solely on structured clinical variables have inherent limitations in predicting post-transplant outcomes. In clinical practice, unstructured data – such as CT imaging and radiology reports – are routinely considered during living donor kidney transplantation (LDKT) evaluations. However, traditional unimodal models often exclude these data types, potentially omitting critical predictive information. For example, recent studies have demonstrated that integrating unstructured data such as free-text clinical notes or imaging can significantly improve predictive performance across various clinical applications, including kidney injury and transplant outcomes^[32–34]^. These findings underscore the limitations of relying exclusively on structured variables. By integrating diverse data modalities, our multimodal deep learning model seeks to overcome these constraints, providing a more comprehensive and accurate assessment of graft function in LDKT recipients.

We hypothesized that this integrative approach would enhance prediction accuracy relative to unimodal models and yield interpretable insights into the relative contributions of each modality. Our study not only presents a novel AI-driven framework for transplant outcome prediction but also provides a foundation for clinical translation by identifying key preoperative factors associated with optimal graft function.

## Methods

### Study population and data collection

**Figure 1.**
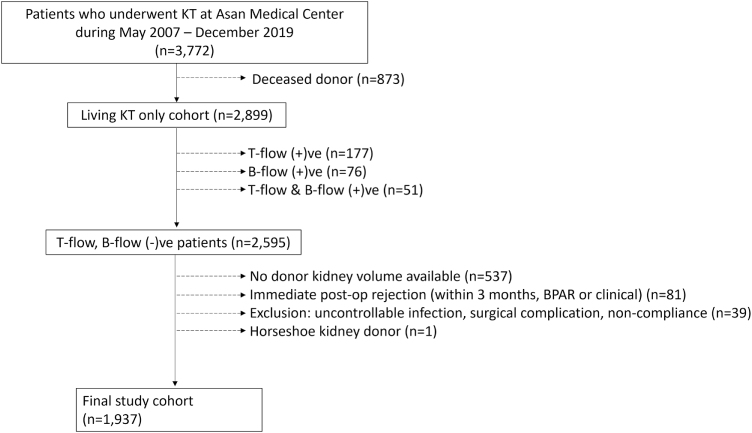
Study cohort flow diagram for model development and analysis.

This retrospective study included patients who underwent kidney transplantation at a single tertiary medical center between May 2007 and December 2019. From an initial cohort of 3772 patients, 873 deceased donor KT recipients were excluded, resulting in 2899 LDKT recipients. Among these, 304 patients were excluded due to immunologic high risk, defined as positive T-cell (*n* = 177), B-cell (*n* = 76), or both T- and B-cell (*n* = 51) flow cytometry crossmatches. This exclusion was intended to restrict the analysis to immunologically low-risk recipients, thereby minimizing confounding from early immune-mediated injury. The remaining 2595 immunologically low-risk LDKT recipients were screened for data completeness. Further exclusions included absence of donor kidney volumetric CT data (*n* = 537), biopsy-proven or clinically suspected acute rejection within the first 3 months post-transplant (*n* = 81), major postoperative complications such as uncontrollable infections, surgical issues, or non-compliance (*n* = 39), and anatomical anomalies such as horseshoe kidney (*n* = 1). After these exclusions, a final cohort of 1937 LDKT recipients with complete clinical, imaging, and follow-up data was included for model development and analysis (Fig. 1). The study is registered at https://clinicaltrials.gov/. This study was reported in accordance with the STROCSS 2025 guideline for cohort studies in surgery to ensure completeness and transparency in reporting^[35]^.

### Multimodal data processing

#### Structured clinical data

Structured variables including demographic information (e.g., age, sex, BMI), transplant-related factors (e.g., HLA mismatch, dialysis duration), and laboratory data were used to construct the tabular dataset (Table 1). Feature preprocessing included target encoding for categorical variables, outlier detection, and missing value imputation^[36]^. These processed features were used as input to tree-based machine learning algorithms.

**Table 1:** Description of the pre-selected features for the model development

Category	Variable name	Description
Recipient factors	Age	Age of the recipient at the time of transplantation
	Sex	Recipient’s biological sex
	t_no	Total number of previous kidney transplants
	Relation	Donor–recipient relationship classification
	BMI	Body mass index of the recipient (kg/m^2^) at surgery
	HD_duration	Duration of pre-transplant hemodialysis (months)
	DM	Diabetes mellitus status of the recipient at time of transplant
	HTN	Hypertension status of the recipient at time of transplant
Donor Factors	Dage	Age of the living kidney donor
	Dsex	Donor’s biological sex
	DDM	History of diabetes mellitus in the donor
	DHTN	History of hypertension in the donor
	DBMI	Donor’s body mass index at time of organ donation
	D_cr	Donor’s baseline serum creatinine level
	D_HbA1c	Glycated hemoglobin (HbA1c) level in the donor
Transplant Factors	abdrMM	Number of mismatches at HLA-A, -B, and -DR loci
	drMM	Number of mismatches at the HLA-DR locus
	dqMM	Number of mismatches at the HLA-DQ locus
	baseCDC	Complement-dependent cytotoxicity (CDC) crossmatch result
	T_flow	T-cell flow cytometry crossmatch result
	B_flow	B-cell flow cytometry crossmatch result
	DSA	Presence of donor-specific anti-HLA antibodies
	ABOi	ABO blood group incompatibility (yes/no)
	Induction	Induction immunosuppressive regimen administered
	DGF	Presence of delayed graft function post-transplant
	Cr_1	Serum creatinine level at 1-year post-transplant
	Cr_2	Serum creatinine level at 2-years post-transplant

#### Text embedding from radiology reports

To convert each radiology report into a usable numeric form, we used a language model (BioBERT), which was pretrained on biomedical corpora including PubMed abstracts and PMC articles, to extract contextual information from all words in the report. These values were then averaged (“mean pooled”) across the entire text to create a single vector summarizing the clinical content of the report for each patient^[37]^. Each radiology report was tokenized and encoded using the BioBERT tokenizer, resulting in 768-dimensional contextual embeddings per report. Token-level representations were aggregated using mean pooling to form a single fixed-length vector representing each patient’s radiological text.

#### CT image preprocessing before embedding extraction

To extract imaging features from donor kidney CT scans, we first compiled sequential axial slices into volumetric video representations for each patient. Frames were uniformly sampled at a fixed temporal rate (every 30 slices) to reduce redundancy and capture representative anatomical information. Each frame was converted from BGR to RGB color space, then preprocessed using the CLIPProcessor from the Hugging Face Transformers library. This step involved resizing to the required input dimensions and normalizing pixel values using the pretrained model’s mean and standard deviation.

#### CT image embedding

For CT imaging, we utilized a CLIP (Contrastive Language–Image Pretraining)-based model for visual feature extraction^[38]^. Each CT video was uniformly sampled to ensure coverage of key anatomical slices. Frames were preprocessed to fit CLIP’s input resolution and passed through the image encoder to produce per-frame embeddings. These were then aggregated using mean pooling to obtain a single, 768-dimensional video embedding per patient, capturing renal morphology and parenchymal characteristics (Supplemental Digital content Figure S1, available at: http://links.lww.com/JS9/F133). Additional implementation details are described in Supplemental Digital content Methods S1, available at: http://links.lww.com/JS9/F133 The purpose of incorporating CT image embeddings was to enable the model to automatically learn imaging-based cues – such as kidney size, cortical thickness, corticomedullary differentiation, or subtle scarring – that may influence graft function but are not represented in structured variables. Rather than preselecting specific imaging variables, the network was allowed to extract latent morphological and parenchymal features from the entire CT dataset, providing complementary information to the clinical and textual inputs.

### Model architecture and integration

**Figure 2.**
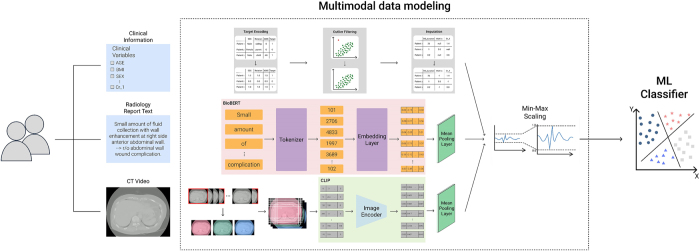
Overview of the proposed multimodal classification framework. The model integrates structured clinical variables, radiology report text, and CT video data. Clinical information is processed as tabular features; radiology text is embedded using a pretrained BERT model; and CT video frames are encoded via a CLIP-based image encoder. Each modality undergoes feature extraction and mean pooling, followed by feature concatenation and min–max normalization. The final fused representation is input into an ML classifier (xGBoost/RF/SVM/LR) to predict the post-transplantation eGFR category.

To predict post-transplantation eGFR levels, we developed a multimodal classification framework that integrates three distinct data modalities: structured clinical information, radiology report text, and CT video data (Fig. 2).

The final multimodal classification model integrated three distinct input modalities: (1) structured clinical data, (2) BioBERT-derived text embeddings, and (3) CLIP-derived CT video embeddings. Each modality underwent independent feature extraction and normalization. To ensure fair comparison across different types of data (e.g., lab values, CT features, text-derived features), we normalized all input values to the same scale using min–max scaling (i.e., rescaling values between 0 and 1). This prevents variables with large numerical ranges from dominating the model. This fused feature space was then used to train supervised classifiers for multiclass prediction of post-transplant eGFR.eGFR was categorized into four clinically relevant strata: > 90, 75–90, 60–75, and 45–60 mL/min/1.73 m^2^. The eGFR categories were derived with reference to CKD staging but modified to suit the expected higher graft function in LDKT recipients. Extremely low eGFR levels (e.g., < 15 mL/min/1.73 m^2^) typical in advanced CKD were not included, as such values are rarely observed in functioning grafts. These adapted thresholds correspond to clinically meaningful strata that inform postoperative care, including the intensity of monitoring, decisions regarding immunosuppressant adjustments, and indications for further diagnostic evaluation. The final predictive task was framed as a multiclass classification problem.

### Model development and algorithms

We implemented and compared four machine learning algorithms for classification:

- eXtreme Gradient Boosting (XGBoost): A gradient-boosted decision tree ensemble incorporating L1/L2 regularization, dynamic tree pruning, and early stopping to prevent overfitting.
- Random Forest (RF): An ensemble of decision trees trained on bootstrapped data with random feature selection, offering robustness and interpretability through feature importance scores.
- Support Vector Machine (SVM): A kernel-based classifier that identifies optimal hyperplanes in a high-dimensional space, particularly effective for nonlinear class boundaries.
- Logistic Regression (LR): A baseline linear classifier extended to the multiclass setting via the softmax function, enabling probabilistic output interpretation.

Model hyperparameters were optimized using nested cross-validation. To evaluate the contribution of each data modality, ablation experiments were conducted using four model configurations: (1) structured clinical data only, (2) clinical data plus radiology text, (3) clinical data plus CT video features, and (4) full multimodal integration. The final predictive framework consisted of an XGBoost classifier trained on a concatenated feature vector integrating (1) structured clinical variables, (2) BioBERT-derived text embeddings from radiology reports, and (3) CLIP-derived embeddings from preoperative CT videos. A complete list of structured variables is provided in Table 1; unstructured data inputs were processed in full, without manual preselection of text or image regions of interest. Performance metrics included macro, micro, and weighted F1 scores.

### Evaluation and interpretability

Model performance was assessed using classification accuracy, sensitivity, specificity, and to assess class-level performance, confusion matrices were generated. Confusion matrices and F1 score breakdowns were used to assess multiclass classification performance for each model variant, with detailed stratification provided for the final multimodal model.

To improve interpretability of the high-dimensional outputs generated by the text and imaging embedding models, we employed dimensionality reduction techniques – *t*-distributed stochastic neighbor embedding (t-SNE) and Uniform Manifold Approximation and Projection (UMAP). These methods project complex, multi-dimensional representations of patient features (e.g., radiologic or textual characteristics) into a two-dimensional space for visualization. This allows for intuitive inspection of clustering patterns among patients, such as whether those with similar graft function outcomes group together in the learned feature space. While these visualizations do not inform clinical decisions directly, they provide supportive insight into how the model internally organizes and differentiates patient profiles based on multimodal input.

To improve transparency and interpretability of the model’s predictions, we applied SHapley Additive exPlanations (SHAP), a model-agnostic technique widely used in machine learning to quantify the contribution of each input feature to a specific prediction. For each patient, SHAP values estimate the directional impact of variables on the predicted eGFR category: positive SHAP values indicate that a variable increases the likelihood of predicting lower graft function, while negative values suggest a protective effect. This enables clinicians to understand which clinical, textual, or imaging features most strongly influenced each individual prediction.

SHAP values were also aggregated to compute global and class-specific importance rankings across all patients, revealing dominant predictors such as donor age, BMI, and donor-recipient sex mismatch. These results help verify that the model’s behavior aligns with known clinical risk factors, while also highlighting additional signal from radiologic and imaging features that are not explicitly represented in structured datasets.

## Results

### Embedding space visualization of multimodal representations

**Figure 3.**
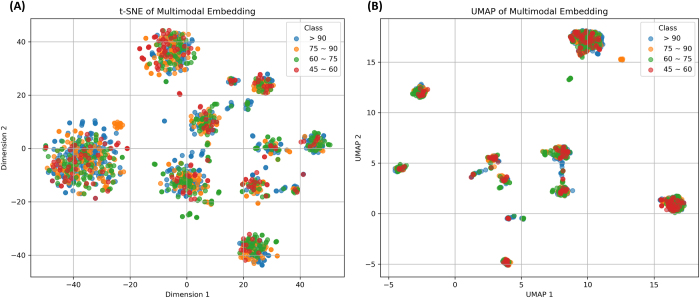
Visualization of multimodal feature embeddings using two-dimensional projection techniques. The fused embeddings obtained from structured, textual, and visual features were reduced to two dimensions using t-distributed stochastic neighbor embedding (t-SNE) (A) and uniform manifold approximation and projection (UMAP) (B) Each point represents a patient sample, color-coded by the corresponding post-transplantation eGFR class (>90, 75–90, 60–75, or 45–60). While both methods reveal local grouping tendencies, substantial overlap among classes is observed. T-SNE emphasizes local clusters within classes, whereas UMAP preserves more of the global structure, enabling visualization of broader inter-class relationships.

To investigate how the model internally organized multimodal information – comprising structured clinical variables, radiology report embeddings, and CT-derived visual features – we visualized the learned feature space using two-dimensional projection techniques: t-SNE and UMAP (Fig. 3 A, B). Each point in the embedding space represents an individual patient, color-coded by their corresponding post-transplant eGFR category: >90, 75–90, 60–75, or 45–60 mL/min/1.73 m^2^.

Both visualization methods demonstrated localized clustering of patients with similar eGFR outcomes, suggesting that the model was able to capture partially discriminative latent patterns across modalities. t-SNE provided more compact local clusters within classes, emphasizing intra-class cohesion, whereas UMAP better preserved the global topological structure, facilitating visualization of broader inter-class relationships. However, substantial overlap remained, particularly between adjacent eGFR categories (e.g., 75–90 and 60–75), reflecting the inherent biological and clinical continuum in renal function outcomes and underscoring the complexity of multimodal feature interactions.

### Incremental modality contribution to predictive performance

To assess the additive value of each data modality, we evaluated the model’s performance across four configurations: (1) structured clinical information only, (2) clinical information with radiological report text, (3) clinical information with CT video embeddings, and (4) full integration of all three modalities. As shown in Table 2, model performance improved progressively with the addition of each modality. While the clinical-only model achieved a macro F1 score of 0.292, this increased to 0.486 with the inclusion of radiological text and to 0.651 with CT video features. The full multimodal model reached the highest macro F1 score of 0.675, micro F1 score of 0.704, and weighted F1 score of 0.698, underscoring the synergistic value of combining structured, textual, and visual data for post-transplant eGFR prediction. This confirms the additive predictive value of radiological data when combined with structured clinical variables. These findings highlight the importance of multimodal integration in capturing the multifaceted determinants of graft function.

**Table 2:** Model performance across data modalities using F1 metrics. Ablation study comparing macro, micro, and weighted F1 scores across four model configurations: (1) structured clinical data only, (2) clinical data plus radiology conclusion text, (3) clinical data plus CT video features, and (4) full integration of all three modalities

	Macro F1	Micro F1	Weighted F1
Clinical data only	0.292	0.361	0.341
Clinical data + Text	0.486	0.515	0.505
Clinical data + Video	0.651	0.668	0.665
Clinical data + Text + Video	0.675	0.704	0.698

### Multiclass classification performance of the optimal model

**Figure 4.**
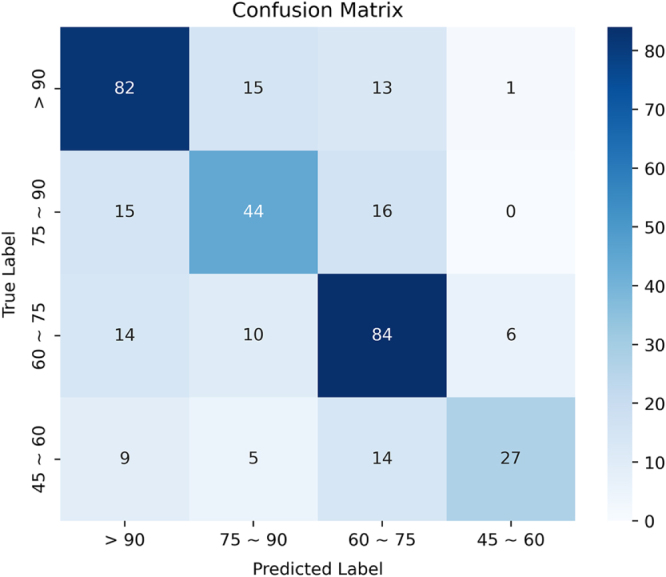
Multiclass classification performance of final model using XGBoost. Confusion matrix summarizing the performance of the final multimodal model trained with XGBoost across four post-transplant eGFR categories: > 90, 75–90, 60–75, and 45–60 mL/min/1.73 m^2^. Precision, recall, and F1-scores are reported for each class. The model achieved the highest precision and recall for the >90 and 60–75 categories, with lower performance for the 45–60 group due to its smaller sample size. Overall macro and weighted F1-scores were 0.675 and 0.698, respectively, indicating strong class-balanced performance. Most misclassifications occurred between adjacent eGFR strata, reflecting clinical overlaps in renal function.

The integration of all three modalities yielded XGBoost as the best-performing model, which was subsequently assessed using a confusion matrix stratified by eGFR categories (Fig. 4). Precision and recall values were highest in the >90 and 60–75 mL/min/1.73 m^2^ categories, with F1 scores of 0.7773 and 0.7303, respectively (Table 3). The model exhibited the lowest recall (0.4182) in the 45–60 group, despite a high precision of 0.8519, indicating a conservative but specific classification pattern in this clinically vulnerable subgroup. The overall accuracy was 0.7042, with a macro F1 score of 0.6754 and weighted F1 score of 0.6982. These results affirm the robustness of the model in distinguishing among clinically meaningful renal function categories, with misclassifications largely confined to adjacent classes – a reflection of the biological continuum in renal function.

**Table 3:** Confusion matrix illustrating the performance of the multiclass classification model across four eGFR categories (>90, 75–90, 60–75, 45–60). Each cell represents the number of samples for which the true class (rows) was predicted as a given class (columns). The model demonstrated high accuracy in distinguishing between normal and moderately impaired kidney function (diagonal values), with most misclassifications occurring between adjacent classes, such as 60–75 vs. 75–90, reflecting the clinical continuity of renal function

Label	Precision	Recall	F1-score	Support
>90	0.7542	0.8018	0.7773	111
75–90	0.6024	0.6667	0.6329	75
60–75	0.6929	0.7719	0.7303	114
45–60	0.8519	0.4182	0.5610	55
Accuracy			0.7042	355
Macro Avg	0.7254	0.6646	0.6754	355
Weighted Avg	0.7176	0.7042	0.6982	355

### Feature importance and modality contributions

**Figure 5.**
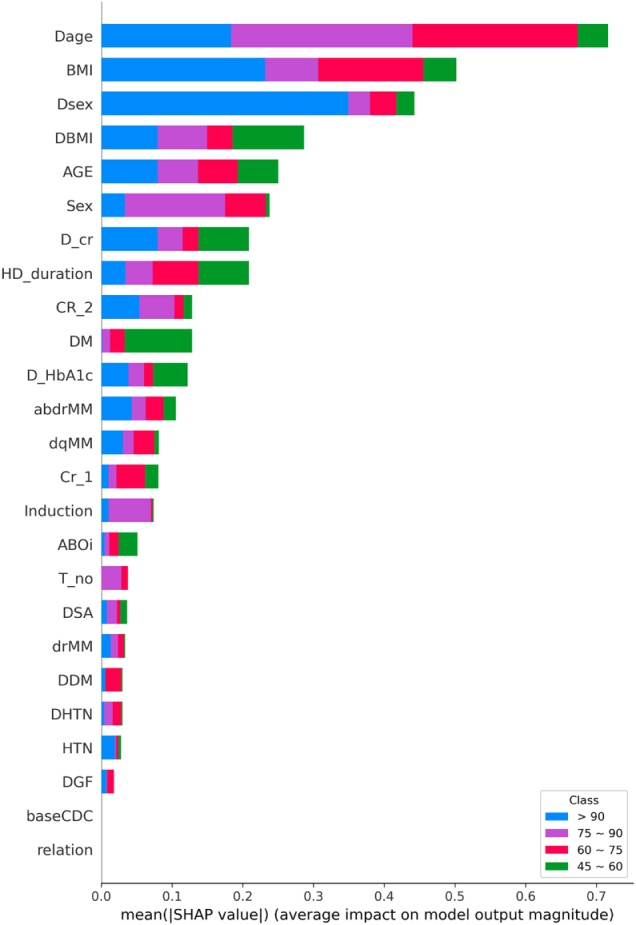
Mean absolute SHAP values for all features, color-coded by class. Donor age (Dage), BMI, and donor sex (Dsex) were consistently influential across all classes. This multi-class SHAP summary reveals both shared and class-specific key predictors.

To interpret model predictions and quantify the relative contributions of each data modality, SHAP analysis was conducted. As illustrated in Figure 5, the SHAP summary plot presents the relative importance of individual structured clinical features when used alone for model prediction. It highlights the top predictors across all eGFR classes, with donor age, body mass index (BMI), and donor sex emerging as the most influential variables in driving the model’s decisions based on structured data alone.

### Class-specific interpretability analysis

**Figure 6.**
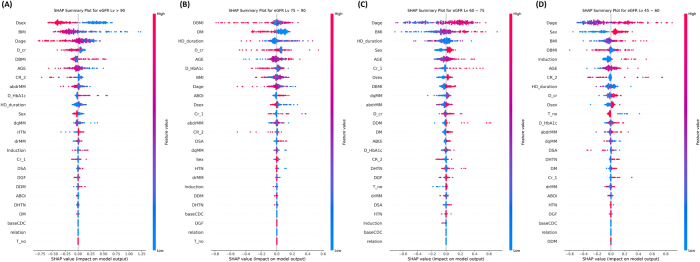
Class-specific SHAP summary plots for predicting each eGFR category: (A) > 90, (B) 75–90, (C) 60–75, and (D) 45–60. Each plot displays the top contributing features for the corresponding class, with SHAP values representing the magnitude and direction of each feature’s influence on the model output. While certain features such as Dage, BMI, and D_cr consistently appear across classes, other features demonstrate class-specific importance. For example, Dsex and AGE were more predictive of Class (A), whereas DM and DBMI were prominent in Class (B), and Induction appeared more influential in Class (D). These findings highlight both shared and distinct patterns in multimodal predictors of kidney function.

Further class-specific SHAP analysis (Fig. 6) revealed nuanced shifts in feature importance across different eGFR strata. In the highest eGFR group (>90 mL/min/1.73 m^2^), favorable graft outcomes were most strongly associated with younger donor age and lower BMI (Fig. 6-A). The 75–90 group (Fig. 6-B) showed similar trends, with donor sex and diabetes status further influencing predictions. For the 60–75 group (Fig. 6-C), the contribution of recipient-related factors – including longer dialysis duration and metabolic comorbidities – became more prominent. Finally, the lowest GFR category (45–60 mL/min/1.73 m^2^; Figure 6-D) was predominantly characterized by high donor BMI, older donor age, and diabetes, highlighting the compounded effect of suboptimal donor-recipient metabolic matches on post-transplant renal function. These results suggest that distinct clinical phenotypes underlie different post-transplant renal function outcomes, and that their effects vary across the spectrum of graft performance. Moreover, while text and video embeddings do not provide interpretable single variables, their inclusion improved model performance, indicating that latent descriptors within radiology reports and CT images – such as cortical thickness, parenchymal quality, and vascular variants – added predictive value beyond structured data.

*Binary Classification Performance*

We additionally evaluated our multimodal model’s ability to predict binary post-transplant renal function status (eGFR > 60 mL/min/1.73 m^2^ vs. ≤ 60 mL/min/1.73 m^2^).

The XGBoost model achieved an AUC of 0.856, sensitivity of 0.81, and specificity of 0.78. Other models tested (Random Forest, Support Vector Machine, and Logistic Regression) yielded AUCs of 0.797, 0.785, and 0.806, respectively (Supplemental Digital content Figure S2, available at: http://links.lww.com/JS9/F133).

### Multiclass ROC analysis

We also assessed model performance across all four eGFR strata using a One-vs-Rest (OvR) classification framework. The class-specific AUCs were as follows: >90: 0.91, 75–90: 0.85, 60–75: 0.87, and 45–60: 0.90, indicating strong discriminative capacity across the full clinical spectrum of post-transplant renal function (Supplemental Digital content Figure S3, available at: http://links.lww.com/JS9/F133).

## Discussion

This study demonstrates that the integration of structured clinical data with CT imaging data and radiological text significantly enhances the prediction of post-transplant renal function in LDKT. By leveraging a multimodal deep learning framework, we were able to incorporate heterogeneous data sources into a unified predictive model that classifies post-transplant eGFR with high accuracy and interpretability. This approach enables personalized prediction of renal function before LDKT, allowing for individualized risk assessment and identification of target eGFR thresholds as indicators of post-transplant prognosis and graft stability.

Although our multimodal model improved overall predictive performance, t-SNE and UMAP plots revealed overlap between adjacent eGFR categories, particularly between 60–75 and 75–90 mL/min/1.73 m^2^. This reflects the biological and measurement continuity of graft function and highlights that the model, while effective at separating extremes, may capture broad trends rather than highly granular distinctions. Future work could explore alternative stratification schemes, regression-based modeling, or additional domain-specific features to enhance discrimination within these intermediate ranges.

The analysis of feature importance via SHAP reinforced the dominant role of structured clinical data in graft function prediction. Donor age, BMI, and diabetes mellitus status consistently emerged as the most influential predictors across all eGFR classes. These findings align with prior literature emphasizing the significance of donor-related factors in post-transplant outcomes, including studies by Kawakita et al^[39]^ and Ali et al^[31]^, which demonstrated the predictive value of donor age and metabolic profile on DGF and long-term survival. Additionally, a study by Siedlecki et al highlighted that DGF is associated with decreased long-term graft survival, with donor age being a significant contributor^[40]^. Similarly, research by J.W. Alexander et al indicated that kidneys from donors over the age of 55 have reduced functional reserve, adversely affecting long-term function^[41]^. Class-specific SHAP analysis in our study revealed that optimal graft function (>90 mL/min/1.73 m^2^) was predominantly associated with younger donor age and lower BMI. Conversely, impaired function (45–60 mL/min/1.73 m^2^) correlated with advanced donor age and higher BMI. These findings are consistent with previous research indicating that both donor and recipient BMI are significant risk factors for DGF and overall transplant outcomes^[42]^. Although class-specific SHAP analyses appear to highlight different dominant features for each eGFR stratum, these differences reflect the relative contribution of the same global predictors (e.g., donor age, BMI, donor sex) in steering predictions toward a particular class. For instance, younger donor age strongly supports classification into the >90 mL/min/1.73 m^2^ group, whereas older donor age contributes more prominently to predictions in the 45–60 mL/min/1.73 m^2^ group. Thus, the observed variation does not imply distinct predictors but rather context-dependent effects of the same underlying factors. Clinically, this reinforces the idea that traditional risk factors may differentially influence stratification depending on the graft function trajectory, underscoring the value of individualized risk interpretation rather than rigid threshold-based assessments.

In this study, the integration of CT imaging features and radiological text embeddings with structured clinical data significantly improved model performance, reinforcing the value of a multimodal deep learning approach in predicting renal outcomes. Structured clinical variables remained the dominant contributors to predictive accuracy – consistent with prior literature and our earlier work, which demonstrated that clinical factors such as T-cell flow cytometry crossmatch, creatinine at 2 years post-transplant, donor sex, and HLA mismatches were the most influential predictors of long-term graft survival using machine learning models that excluded imaging data^[43]^. The addition of imaging and textual features in the present study provided complementary, non-redundant information that enhanced the model’s discriminative power, particularly for intermediate-risk categories (eGFR 60–75 and 75–90 mL/min/1.73 m^2^). This observation aligns with findings in the broader field of medical imaging AI, where studies have shown that models combining imaging data with clinical metadata often achieve enhanced diagnostic accuracy^[44–46]^. A recent study by Jeon et al^[29]^ developed a high-performing machine learning model to predict post-donation eGFR in living kidney donors using a comprehensive set of structured clinical and imaging-derived variables. Our study complements this work by extending predictive modeling into the post-transplant recipient setting and exploring the added value of integrating multimodal inputs –structured data, radiology report text, and CT images – into a unified framework. This approach may help capture subtle morphologic and contextual cues that are not routinely encoded in structured fields and thus improve prediction of long-term graft function. The modest yet meaningful contributions of imaging and textual modalities in our study underscore their role as context-enriching inputs that enhance model robustness and generalizability. This suggests that although the incremental performance gains from imaging were modest compared to structured data, these modalities offered additional granularity by capturing anatomical and textural variations not represented in clinical variables. For instance, CT-derived features extracted via CLIP-based video embeddings were particularly helpful in delineating mid-range renal function categories, which are often susceptible to under-recognized subclinical deterioration. Although the text and imaging data are processed as high-dimensional embeddings, interpretability is enhanced through SHAP analysis, which revealed that structured clinical variables – particularly donor age, BMI, and donor sex – contributed the greatest share of predictive power. Meanwhile, the inclusion of radiology and CT embeddings allowed the model to capture semantic and morphological information that is complementary to structured data. This integration enabled improved discrimination, particularly in intermediate-risk patients, thereby justifying the inclusion of all three modalities. These results highlight the importance of multimodal AI in capturing the complex interplay between structural, functional, and clinical attributes that influence graft performance. Multimodal learning enhances model generalizability by reducing overfitting to specific feature types and increasing robustness across diverse patient phenotypes. This integrative framework not only refines risk stratification but also lays the groundwork for developing personalized decision-support systems in the filed of transplantation medicine. The incorporation of multimodal data further reflects the complex interplay of anatomical, functional, and clinical factors influencing graft outcomes. By leveraging the strengths of each data type, the model offers a more comprehensive assessment – particularly valuable for nuanced risk stratification among transplant recipients.

While the macro F1 score of 0.675 does not meet the threshold typically sought for clinical deployment, it represents a substantial improvement over baseline performance (macro F1 = 0.292 using clinical data alone), underscoring the added value of integrating imaging and textual data. In the context of complex transplant prediction tasks, this performance level offers a meaningful step toward personalized risk stratification.

Taken together, our results reinforce the growing consensus that multimodal AI can outperform unimodal approaches by leveraging complementary strengths across data types. The modest yet meaningful contributions of imaging and textual modalities underscore their role not as primary predictors, but as essential context-enriching inputs that enhance model robustness and generalizability – particularly when clinical data alone are insufficient to capture the full biological complexity of graft function outcomes.

While the proposed model demonstrates robust classification performance, several limitations must be acknowledged. First, the retrospective design introduces the possibility of selection bias and unmeasured confounders. Second, although the model captured high-dimensional interactions among input modalities, the overlap observed in t-SNE and UMAP plots suggests that further refinement is necessary to capture complex latent features. Third, the relatively limited contribution of imaging and textual data – despite the use of advanced embedding techniques – suggests that current imaging modalities may not yet effectively capture the functional characteristics or physiological integrity of the transplanted graft. To overcome these limitations, future research should prioritize the incorporation of longitudinal follow-up data such as serial eGFR, doppler ultrasound metric, or protocol biopsy findings. Moreover, the dataset used in this study was derived from a single tertiary center, and model performance was evaluated using internal cross-validation only. External validation on independent cohorts was not performed, partly due to the logistical and technical challenges of multimodal data harmonization across institutions. While these constraints limit generalizability, the model’s performance within the current multimodal framework still demonstrates proof-of-concept feasibility. Future studies should aim to standardize multimodal pipelines to enable scalable external validation and cross-center adaptation. From a translational perspective, the development of clinician-facing decision-support tools based on interpretable AI frameworks may facilitate personalized donor selection, risk stratification, and post-transplant monitoring. Although some overlap between adjacent eGFR categories is expected due to the biological continuum of renal function, the observed persistence of class intermingling in low-dimensional embeddings suggests that the latent space learned by the model may not be fully optimized for fine-grained classification. This limitation reduces confidence in the model’s use for precise, individualized decision-making in borderline cases. Future work could explore alternative modeling strategies – such as regression approaches, ordinal classification frameworks, or domain-specific feature engineering – to better structure the latent space and enhance discrimination within clinically challenging intermediate ranges.

While our study demonstrates the feasibility and predictive benefit of integrating radiology text embeddings and CT image embeddings, we acknowledge that implementing these pipelines in routine practice poses logistical and technical challenges, including access to raw imaging data, preprocessing workflows, and AI-specific infrastructure. These requirements may limit immediate adoption in centers without dedicated bioinformatics support. Future work should focus on developing simplified, standardized embedding pipelines and exploring centralized or cloud-based solutions to facilitate broader clinical deployment. Morever, while structured clinical variables accounted for the majority of predictive performance in our study, the integration of radiology text and imaging embeddings provided incremental gains, particularly in mid-range eGFR strata where clinical data alone were less discriminative. We acknowledge that these gains may not justify the added complexity in resource-constrained settings; however, demonstrating the feasibility and added value of multimodal approaches provides a foundation for future development and selective application in contexts where enhanced precision is clinically meaningful. Although our multimodal model improved prediction metrics compared with clinical variables alone, it still misclassified approximately 30% of cases across clinically meaningful eGFR thresholds. Such misclassifications, if directly acted upon, could lead to inappropriate levels of post-transplant monitoring or even incorrect acceptance or exclusion of donor–recipient matches. We therefore emphasize that these results represent a methodological proof of concept rather than a deployable clinical decision tool.

In conclusion, this study presents a novel and interpretable multimodal machine learning framework for predicting post-transplant renal function in LDKT recipients. The findings emphasize the predominant predictive value of clinical variables while highlighting the potential for augmented accuracy through complementary imaging and text-based data. As transplant medicine moves toward precision care, the integration of multimodal data analytics offers a promising path forward in optimizing graft outcomes and individualizing patient management.

## Supplementary Material

## Data Availability

The datasets generated and/or analyzed during the current study are available from the corresponding author upon reasonable request.

## Research registration unique identifying number (UIN)

The study is registered at https://clinicaltrials.gov/study/NCT06394596.
