# Supplementary material for: Multimodal deep learning integration for predicting renal function outcomes in living donor kidney transplantation: a retrospective cohort study
Source: Int J Surg. 2025 Sep 17;112(1):1153–63. doi: 10.1097/JS9.0000000000003494 (PMC12825729; doi:10.1097/JS9.0000000000003494)
Supplement: Supplementary file 1 [file js9-112-1153-001.docx]

**Supplementary materials**

**Methods S1.**

**CT Imaging Preprocessing and Embedding Extraction Pipeline**

To incorporate donor kidney morphological features into our multimodal prediction model, we developed a standardized pipeline to preprocess preoperative abdominal CT scans and extract imaging features using a vision-language transformer model (CLIP). The entire procedure was implemented in Python (v3.9.13) using OpenCV (v4.8.0), Hugging Face Transformers (v4.33.1), and PyTorch (v2.0.1). The following steps describe the complete pipeline from raw DICOM images to the final 768-dimensional imaging embedding per donor.

**1. Input Data Structure and Formatting**

Donor CT data were acquired as standard DICOM series, consisting of sequential axial slices obtained from non-contrast abdominal scans performed for pre-transplant donor evaluation. Each patient’s scan spanned the entire kidney volume and included at least 120–200 slices per scan with variable slice thickness (typically 2.5–5 mm).

To prepare this data for temporal sampling, we:

- Extracted pixel matrices using the pydicom library (v2.3.1).
- Rescaled pixel intensities using the rescale slope/intercept from DICOM headers.
- Ordered slices based on the ImagePositionPatient metadata to preserve anatomical continuity.
- Stacked all slices into a 3D NumPy array of shape (H, W, D), where H = height, W = width, and D = number of slices.

**2. Volumetric Video Construction and Frame Sampling**

To capture 3D anatomical structure in a form suitable for 2D visual encoders, we converted axial slices into sequential RGB video frames.

- Each slice was duplicated across three channels (to simulate RGB) and saved as PNG frames.
- A sampling rate of 1 frame every 30 slices was used to downsample each scan, resulting in ~5–7 representative frames per CT volume. This rate was selected empirically to balance anatomical diversity and computational efficiency.

The goal of this step was to avoid over-representation of redundant adjacent slices and instead preserve coarse-to-fine anatomic variations (upper pole to lower pole), including renal hilum, cortex, medulla, and perirenal fat.

**3. Preprocessing with CLIPProcessor**

Each sampled frame was preprocessed using the CLIPProcessor utility from Hugging Face’s Transformers library, compatible with the CLIP ViT-B/32 visual encoder pretrained on the LAION-400M dataset. Preprocessing included:

- Color space conversion from OpenCV BGR to RGB.
- Image resizing to 224 × 224 pixels using bilinear interpolation.
- Pixel normalization using CLIP’s pretrained mean and standard deviation:
  - Mean: [0.48145466, 0.4578275, 0.40821073]
  - Std: [0.26862954, 0.26130258, 0.27577711]

These statistics were derived from the distribution of pixel intensities in the original CLIP training corpus and are critical for maintaining feature compatibility.

Preprocessing was applied via:

from transformers import CLIPProcessor, CLIPModel

processor = CLIPProcessor.from_pretrained("openai/clip-vit-base-patch32")

inputs = processor(images=sampled_frames, return_tensors="pt")

4. Embedding Extraction Using CLIP Visual Encoder

Following preprocessing, frames were passed through the CLIP visual encoder (ViT-B/32) as follows:

- Each image produced a 768-dimensional feature vector corresponding to the final token from the vision transformer output.
- Frame-level embeddings were extracted in batch mode using:

model = CLIPModel.from_pretrained("openai/clip-vit-base-patch32")

with torch.no_grad():

outputs = model.get_image_features(**inputs)

5. Postprocessing and Aggregation

To ensure consistent feature scaling and facilitate fusion with other modalities, we performed the following:

- L2 normalization of each frame-level vector:


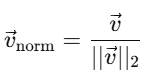


- Mean pooling across all frames per patient:


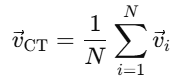


where N is the number of sampled frames and v⃗i\vec{v}_ivi​ is the embedding for frame *i*.

This aggregation yielded a single normalized 768-dimensional vector representing the donor’s overall renal morphology and anatomical variation, which was concatenated with other modality embeddings (clinical and text) for multimodal prediction.

**6. Justification of CLIP-Based Embedding Approach**

The rationale for using CLIP embeddings lies in their capacity to encode high-level visual semantics and spatial relationships learned from large-scale vision-language corpora. Although not fine-tuned on medical images, CLIP has demonstrated robust transfer learning performance across domains. By using pretrained vision transformers without task-specific fine-tuning, we aimed to capture latent morphological information (e.g., renal contour, parenchymal thickness, corticomedullary differentiation) in a modality-agnostic manner.

**7. Code Reproducibility**

All image preprocessing, embedding extraction, and aggregation steps were implemented using open-source Python packages and are available upon reasonable request. The code supports GPU acceleration and batch inference and is compatible with PyTorch-based multimodal modeling frameworks.


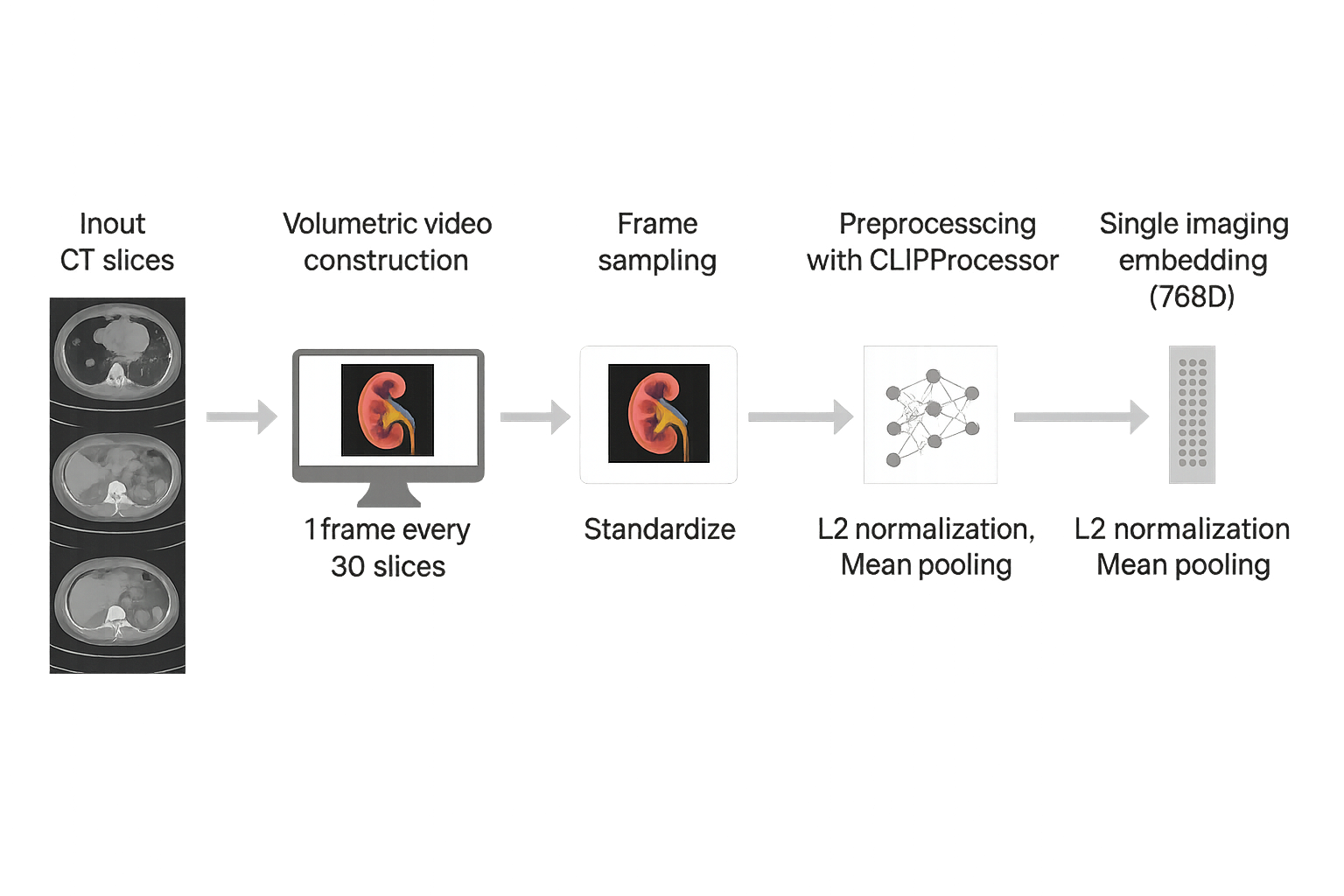


**Figure S1. Preprocessing Pipeline for Donor CT Image Embeddings Using CLIP.** This diagram illustrates the preprocessing workflow applied to preoperative donor kidney CT scans for feature extraction using a CLIP-based visual encoder. Sequential DICOM images were compiled into CT video files. Uniform frame sampling was performed (sampling rate = 30) to reduce redundancy and capture representative anatomical information. Each sampled frame underwent color space conversion (BGR → RGB) and image normalization via the CLIPProcessor, which included resizing and standardization based on model-specific mean and standard deviation. Finally, frame-level feature embeddings were extracted using the CLIP encoder and aggregated using mean pooling, followed by L2 normalization to generate a fixed-length feature vector for each patient.

**
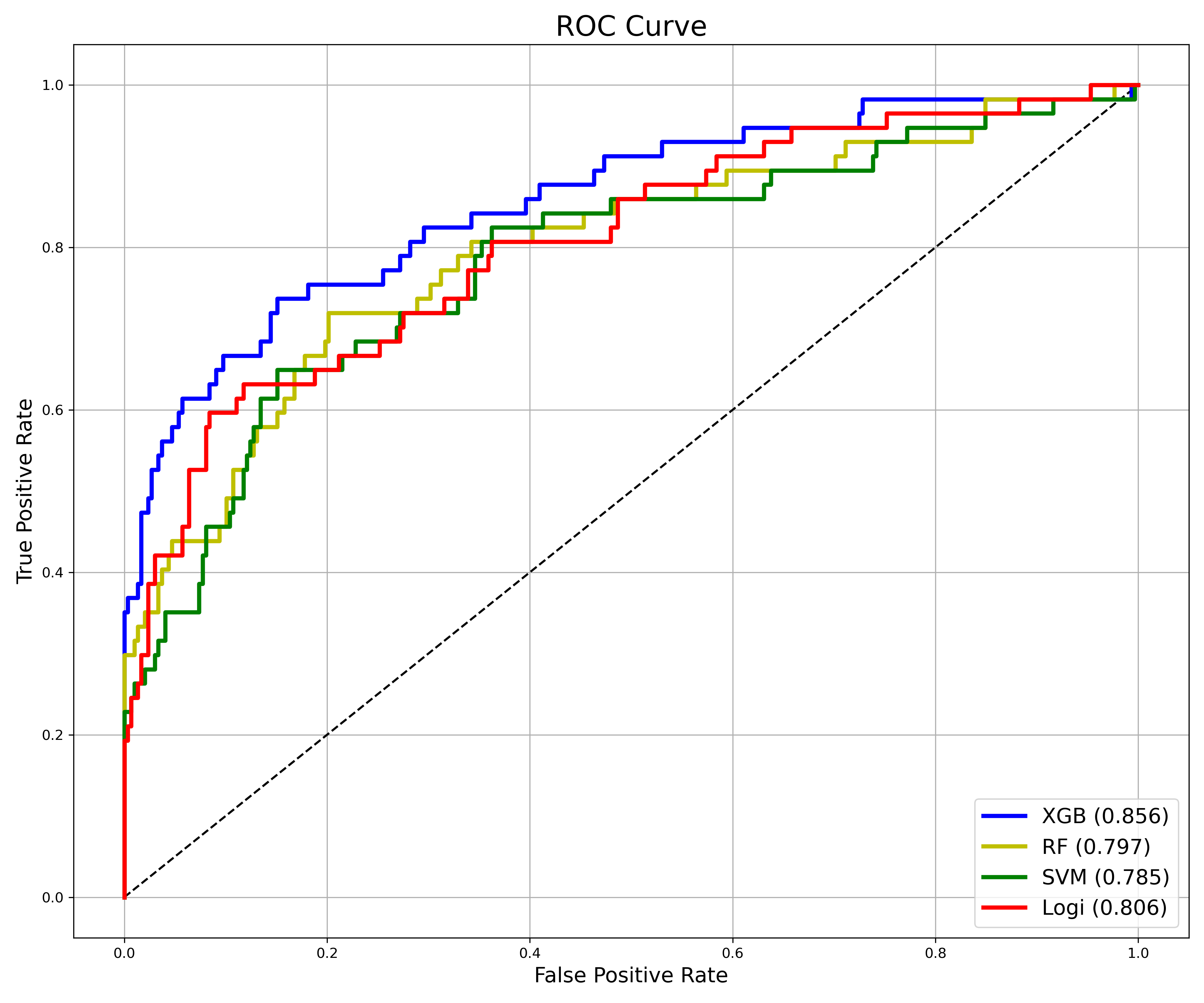
**

**Figure S2. ROC Curve for Binary Classification (eGFR > 60 vs. ≤ 60).** Receiver operating characteristic (ROC) curves for predicting adequate post-transplant renal function (eGFR > 60 mL/min-1.73m²). This figure compares the performance of four machine learning classifiers: XGBoost (AUC = 0.856), Random Forest (AUC = 0.797), Support Vector Machine (AUC = 0.785), and Logistic Regression (AUC = 0.806).

**
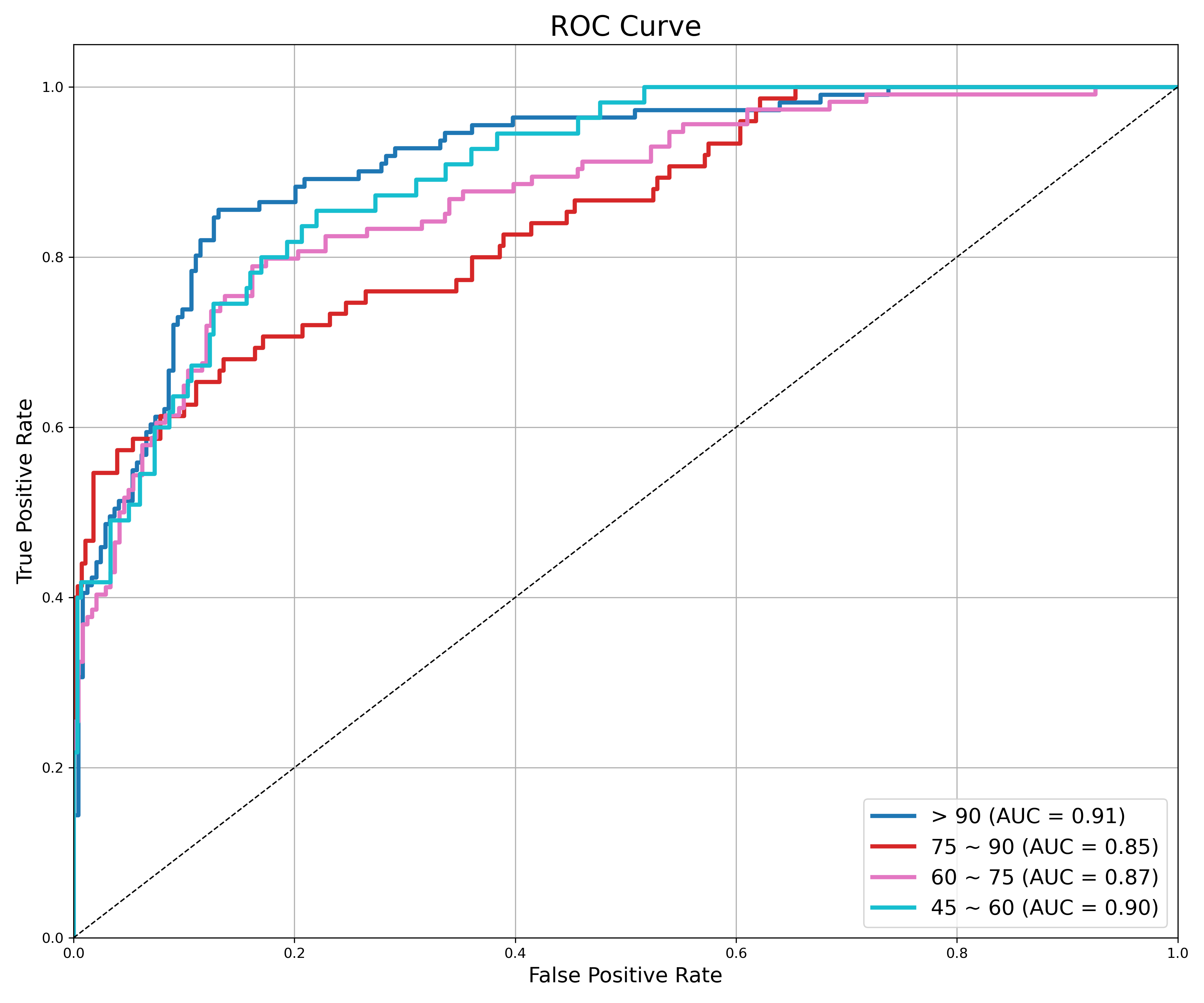
**

**Figure S3. One-vs-Rest ROC Curves for Multiclass classification (eGFR strata).** This figure illustrates the performance of the final multimodal XGBoost model for the four-class eGFR prediction task, evaluated using a One-vs-Rest (OvR) strategy. Each curve represents the model's ability to distinguish a single eGFR category from all other categories combined. The Area Under the Curve (AUC) for each class is as follows: >90 (AUC = 0.91), 75–90 (AUC = 0.85), 60–75 (AUC = 0.87), and 45–60 (AUC = 0.90). These high AUC values across all strata demonstrate the model's robust discriminative capability for each clinically defined renal function outcome.
